# Effects of the Simultaneous Presentation of Corresponding Auditory and Visual Stimuli on Size Variance Perception

**DOI:** 10.1177/2041669518815709

**Published:** 2018-12-11

**Authors:** Sachiyo Ueda, Ayane Mizuguchi, Reiko Yakushijin, Akira Ishiguchi

**Affiliations:** Department of Computer Science and Engineering, Toyohashi University of Technology, Japan; Graduate School of Humanities and Sciences, Ochanomizu University, Japan; Department of Psychology, Aoyamagakuin University, Japan; Faculty of Core Research, Ochanomizu University, Japan

**Keywords:** crossmodal correspondence, ensemble perception, multisensory processing, variance perception

## Abstract

To overcome limitations in perceptual bandwidth, humans condense various features of the environment into summary statistics. Variance constitutes indices that represent diversity within categories and also the reliability of the information regarding that diversity. Studies have shown that humans can efficiently perceive variance for visual stimuli; however, to enhance perception of environments, information about the external world can be obtained from multisensory modalities and integrated. Consequently, this study investigates, through two experiments, whether the precision of variance perception improves when visual information (size) and corresponding auditory information (pitch) are integrated. In Experiment 1, we measured the correspondence between visual size and auditory pitch for each participant by using adjustment measurements. The results showed a linear relationship between size and pitch—that is, the higher the pitch, the smaller the corresponding circle. In Experiment 2, sequences of visual stimuli were presented both with and without linked auditory tones, and the precision of perceived variance in size was measured. We consequently found that synchronized presentation of audio and visual stimuli that have the same variance improves the precision of perceived variance in size when compared with visual-only presentation. This suggests that audiovisual information may be automatically integrated in variance perception.

## Introduction

There are many kinds of diversity in the world, such as various types of species, ideas, colors, shapes, textures, and sounds. Even elements within the same category show variations. For example, when buying packed fruit, one package may contain items that are all approximately the same size, while others may contain items that vary in size. In this case, uniform-sized fruit may represent good quality and, consequently, the buyer may be more likely to choose the former package. In another example, when a group of people with different opinions regarding a matter finally arrive at a single conclusion, there may be considerable variations in the people’s facial expressions (i.e., emotions), ranging from being very satisfied to very dissatisfied with the conclusion. In such cases, it might be necessary to pay additional attention to the opinions of each member.

To understand the outside world, information about variations in objects and creatures is useful and can affect subsequent decision-making and behaviors. Therefore, it is important to clarify the mechanisms through which humans perceive variations. Quantitative variation within the same category can be treated as variance. Performing variance and averaging when encountering a population allows an individual to discern representative values for that population; in other words, summary statistics are determined, and many researchers have demonstrated that humans can perceive summary statistics very efficiently (e.g., Haberman, Lee, & Whitney, 2015; Morgan, Chubb, & Solomon, 2008; Solomon, 2010; Solomon, Morgan, & Chubb, 2011).

Compared with studies on variance perception, more studies have been performed on averaging, such as in regard to orientation (e.g., Parkes, Lund, & Angelucci, 2001; Robitaille & Harris, 2011), size (e.g., Ariely, 2001; Attarha, Moore, & Vecera 2014; Chong & Treisman, 2003), luminance (e.g., Bauer, 2009), length (Weiss & Anderson, 1969), speed (e.g., Emmanouil & Treisman, 2008; Watamaniuk & Duchon, 1992), direction of motion (e.g., Watamaniuk, Sekuler, & Williams, 1989), facial expressions (Haberman, Harp, & Whitney, 2009; Haberman & Whitney, 2009), and gender (Haberman & Whitney, 2007). Averaging is not limited to the spatial dimension; it can be applied to stimuli that change in size over time (Albrecht & Scholl, 2010) and can even be applied in perceptual modalities other than visual. For example, in auditory perception, it is possible to create an accurate average perception of pitch (Albrecht, Scholl, & Chun, 2012; Piazza, Sweeny, Wessel, Silver, & Whitney, 2013); Albrecht et al. (2012) also reported that the observer showed little cost in computing the averages of tone and visual size presented simultaneously. Through averaging, humans can efficiently perceive the gist of groups that have redundant information; this means that they can effectively reduce the influence of the intrinsic noise associated with each stimulus and accurately grasp the content of the whole (Alvarez, 2011).

On the other hand, variance does not describe the central tendency of a stimulus set; it represents the overall distribution pattern instead. It conveys general information about population characteristics and the reliability of averages of certain qualities (such as those described earlier); in some cases, a large variance in a system may also indicate abnormalities and potential risks (Ueda, Yakushijin, & Ishiguchi, 2015). Thus, determining both variance and average is important for efficiently understanding environmental information and executing appropriate adaptive behavior. Previous studies have shown that variance perception of visual features, such as orientation (e.g., Morgan et al., 2008) and size (e.g., Solomon et al., 2011), is performed efficiently. For instance, Solomon et al. (2011), through an ideal observer analysis using a computational process model, showed that variance perception is more accurate and efficient than average perception. They also argued that late noise, which has been theorized to affect average perception, does not affect variance perception. Other studies have shown that for higher order stimuli that have social significance, such as facial expressions, variance can be perceived accurately and efficiently, even if individual face perception fails at the conscious level (Haberman et al., 2015). Meanwhile, regarding auditory stimuli, it has been found that humans can discriminate between variances in rhythm (e.g., empty time intervals marked by auditory tones; Ashitani, Yakushijin, & Ishiguchi, 2012); in this study (Ashitani et al., 2012), just noticeable differences for several standard auditory stimuli were fitted to dipper functions and were determined to be identical to characteristics reported in variance discrimination of visual stimuli (orientation; Morgan et al., 2008).

Although most research on variance perception has focused on single modalities, the human brain uses multiple sources of sensory information derived from several different modalities to reconstruct the external environment. By merging these different sources of information efficiently, humans can develop a coherent and robust perception from noisy unisensory perceptual estimates (Ernst & Bülthoff, 2004). Considering this limitation of previous studies, it is clearly necessary to examine variance perception based on information from multiple modalities. For example, when encountering elephant herds, the variance is discerned using both visual and auditory information, because big elephants make low-frequency vocal sounds and small elephants make high-frequency vocal sounds. That is, visual information regarding the elephants’ individual sizes and auditory information regarding their vocal frequencies are correlated with high probability. By integrating auditory information (pitch) that is linked with visual information (size), the perceived signal regarding visual size may become more salient, and an increase in the saliency of an individual stimulus affects perception of the entire stimulus set. Generally, if salience produced by multimodal presentation reduces the noise (e.g., Gaussian noise) in the internal representation of the stimulus components, the reduction effects will be larger on the statistical variance than on the statistical mean of the stimulus values. Thus, in this study, we investigate the crossmodal effect especially on variance perception.

Many studies have shown that several nonarbitrary associations appear to exist between basic physical attributes of stimuli or features of different sensory modalities (e.g., Martino & Marks, 1999; Melara & O’Brien, 1987; Parise & Spence, 2009; Spence, 2011). Crossmodal correspondence is partially similar to synesthesia but does not necessarily show personal inherent ties between sensory modalities and the conscious experience of additional sensory features. Crossmodal correspondence concerns a consistent combination of multisensory features, and it has been reported in many people (without synesthesia). There are links between different features in various levels of processing, such as perceptual or semantic levels. For example, there are associations between shape and phonology, as shown by the *bouba/kiki* effect (Ramachandran & Hubbard, 2001, 2003); moreover, perception of the motion direction of ambiguous visual-motion displays has been found to be affected by a crossmodally corresponding sound (rising or falling) when it is presented simultaneously at the onset of the visual-motion stimulus (Maeda, Kanai, & Shimojo, 2004).

On the basis of this dependency between modalities, we infer that effective sampling exists in crossmodally corresponding presentations. There are two possibilities by which crossmodal presentation can make sampling effective. First, it is possible that being presented with a corresponding sound can increase the salience of the visual stimulus. Even when a signal in the primary modality is not sufficiently salient to be sampled, the corresponding signal from another modality can strengthen it and make it suitable for sampling. Through this process, the sample size may be increased or stabilized, thereby leading to good variance perception. Second, crossmodal correspondence has been determined to promote efficient information processing (e.g., Evans & Treisman, 2010; Gallace & Spence, 2006; Marks, 1987; Martino & Marks, 1999; Melara & O’Brien, 1987). In a speeded classification task in which participants are asked to decide which of two successively presented stimuli (e.g., circles) are larger, the response time is shorter when a crossmodally congruent sound (e.g., a high-pitch sound to accompany a small circle) is presented with the second stimulus than when a crossmodally incongruent sound (e.g., a high-pitch sound to accompany a large circle) is presented (Evans & Treisman, 2010; Gallace & Spence, 2006). Such improvement in efficiency has been shown between pitch and visual stimuli, including height (e.g., Evans & Treisman, 2010; Melara & O’Brien, 1987), brightness (Marks, 1987), shape and angularity (Marks, 1987), and spatial frequency (Evans & Treisman, 2010). However, there is room for discussion regarding this issue: at which stages of processing do these links appear? Nevertheless, it is very likely that crossmodal correspondence promotes perceptual processing and variance perception.

Based on the aforementioned findings, this study examined whether the simultaneous presentation of corresponding auditory stimuli facilitates the discrimination of visual variance. To perform this, combinations of visual sizes and auditory pitches for which crossmodal correspondence was reported in prior studies (e.g., Evans & Treisman, 2010; Gallace & Spence, 2006; Parise & Spence, 2009) were used. The following four conditions were set for the combinations of audio-visual stimuli, and the discrimination thresholds of size variance were compared between the conditions: (a) crossmodally congruent condition, (b) crossmodally incongruent condition (the same stimulus set as the congruent condition, but rearranged to avoid presenting crossmodal congruence), (c) constant pitch condition, and (d) no-sound condition. In terms of improving processing accuracy, including the saliency increment described earlier, we hypothesized that the sensitivity of size-variance discrimination would improve only in the congruent condition.

We conducted two experiments to examine this hypothesis. In Experiment 1, we measured, for each participant, their corresponding relationship between visual size and auditory pitch. This experiment had two aims. One was to confirm, through the use of an adjustment method, the link between visual size and auditory pitch described earlier; the other was to determine the corresponding functional equation between the size and pitch for each participant in order to apply it in Experiment 2. Although crossmodal correspondence is common in a relatively large number of people, it is possible that there are individual differences regarding the functional relationship between the two properties (i.e., the pitch that corresponds to a particular size). To efficiently examine the crossmodal-congruency effect on variance perception, it was important to use the optimal combination of size and pitch for each participant; consequently, in Experiment 2, we adjusted the sound stimuli individually for each participant using the functional equations obtained in Experiment 1. In Experiment 2, participants observed two successive sequences, each consisting of eight stimuli (i.e., disks with sounds), and decided which sequence of disks had a larger variance of size. Previous studies using the speeded classification task have shown that the effects of crossmodal correspondence between visual size and auditory pitch are not absolute but relative (Gallace & Spence, 2006; Melara & O’Brien, 1987); when high- or low-pitch sounds are presented in the same block, the relative stimuli values create a congruent effect, but this effect disappears when the high or low sounds are presented in different blocks (Gallace & Spence, 2006). Nevertheless, in the experiments described in this study, since the sizes of the disks and the pitches of the sounds were relative for each stimulus range, this procedure could satisfy the aforementioned condition for generating synesthesia-like effects.

## Experiment 1

### Methods

#### Participants

Participants were 22 paid volunteers (all females, aged 21–29 years). Of these, 19 were graduate students of Ochanomizu University, while the others were undergraduate students of Aoyama Gakuin University. All participants provided written informed consent before the experiment. Twenty-one participants reported having normal or corrected-to-normal vision and no hearing problems; one participant (Participant 20) reported suspecting herself to be tone deaf. Twenty participants had at least 3 years of experience with musical instruments; the remaining two (Participants 4 and 7) had no specific musical experience but had attended music classes through Japanese compulsory education.

#### Apparatus

We generated visual and auditory stimuli using MATLAB with the Psychtoolbox expansion (Brainard, 1997; Pelli, 1997) on a MacBook Pro computer. Visual stimuli were displayed on a 17-in. CRT monitor with a resolution of 1,152 × 864 pixels (refresh rate of 75 Hz). For each observer, auditory stimuli were presented at the same moderate volume via headphones (SONY MDR-CD900ST). Participants sat in a dimly lit room and, using a chin rest, observed the monitor from a distance of approximately 57 cm. For each experimental trial, they pressed a key on an Apple keyboard to give a response.

#### Stimuli

Pure tones of five frequencies (200, 400, 800, 1600, and 3200 Hz) were used as auditory stimuli. In each trial, one of the tone pitches was randomly chosen and presented for 500 ms. For the visual stimulus, a white disk was presented on a gray background in the center of the display. There were two initial values for the disk diameter (a visual angle of 1° or 5°), with one of these randomly chosen and presented in each trial. The disk diameter could be changed by the participants: Each press of the *up arrow* key contracted the disk by a visual angle of 0.1°, and each press of the *down arrow* key expanded it by 0.1°. The luminance of the gray background was 1.22 cd/m^2^ (CIE *xy* chromaticity coordinates, *x* = 0.266, *y* = 0.433), and the luminance of the white disk was 91.4 cd/m^2^ (CIE *xy* chromaticity coordinates, *x* = 0.256, *y* = 0.337).

#### Procedure

The participants were asked to adjust the disk size using the keys so that it matched the pitch of the presented sound (i.e., they applied an adjustment method).

In each trial, a fixation point was presented in the center of the screen, and a pure tone was played through the headphones for 500 ms. The fixation point remained on screen during the tone presentation. Then, after a blank display without sound for 500 ms, a white disk was presented and participants proceeded to adjust the disk size by pressing the keys. When the size adjustment was completed, participants pressed the space key. After this, the original pure tone was presented again for confirmation. If participants judged the disk size to match the sound pitch, they pressed the space key once more; otherwise, they could readjust the disk size before pressing the space key for the second time. The disk size at the time of the second pressing of the space key was recorded as the adjusted size for the trial. The next trial began 500 ms after the second press of the space key.

There were 10 conditions in total (five pitch conditions for each of the two initial disk sizes). Before the experimental session, a trial for each condition was presented in random order as practice. In the experimental session, five trials for each condition were presented randomly; that is, the session comprised 50 trials in total. It took approximately 20 min for each participant to finish this experiment, including the time required to provide experiment instructions and conduct practice trials.

### Results and Discussion

**Figure 1.**
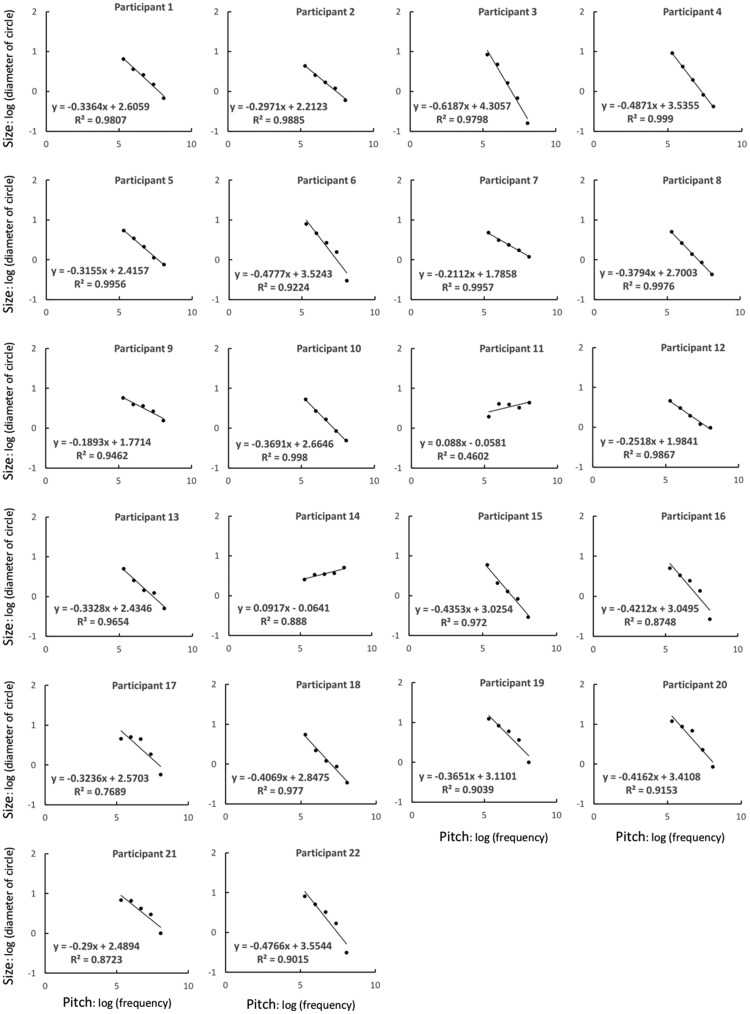
Graphs of the relationship between pitch and size for each participant in Experiment 1.

To examine the perceptual relationship between size and pitch, for each participant, the adjusted disk sizes they reported for each of the five pitch conditions were averaged to give single values for each condition. We plotted the data on logarithmic axes because such axes seem to be more appropriate for examining sensory relationships than are linear axes. The pitch of a sound is proportional to its frequency, and Solomon et al. (2011) showed, in an experiment concerning size discrimination of circles, that a circle’s effective size is proportional to the logarithm of its diameter. Although Solomon et al. also acknowledged other possibilities for this relationship (e.g., suggesting an alternative model of size discrimination that includes Gaussian decision noise that increases with circle size), in the present study we assumed that perceived size corresponds to logarithmically transduced circle diameters. The regression lines on the logarithmic axes for each participant are shown in Figure 1. The lines seem to fit the data well (the *R*^2^ for all participants, except Participants 11, 14, and 17, was 0.87 or higher; although the *R*^2^ of Participant 14 was sufficiently large, their correlation was negative), which demonstrates the linear relationship between pitch and size. This finding suggests that the participants did not adjust the size randomly but had somewhat consistent perceptions of the sizes corresponding to each pitch. In addition, the regression lines for 20 of the participants were downward and to the right. These results show that crossmodal correspondence between size and pitch (i.e., large size to low pitch and small size to high pitch) exists, as was reported in previous studies (Evans & Treisman, 2010; Gallace & Spence, 2006; Parise & Spence, 2008, 2009). Moreover, the differences in the slopes for each participant suggest the existence of individual differences in how size is associated with a certain pitch. For Experiment 2, in order to use stimuli with the strongest possible correspondence between pitch and size, we applied the parameters of the regression lines obtained for each participant in Experiment 1 to the stimulus settings.

In Experiment 1, two participants (Participants 11 and 14) showed a reverse relationship between size and pitch (i.e., large size to high pitch and small size to low pitch). They did not differ from the other participants in terms of musical experience or hearing. Thus, the reason for this inverse perception is unclear, but this result suggests that, although the relationship between size and pitch was consistent in most participants, crossmodal correspondence may partly depend on individual factors. We did not conduct further investigation in this regard, but it may be worthwhile to examine whether people who show such an inverse crossmodal link also differ from others when performing tasks such as those applied in previous related studies.

As shown in Figure 1, the slopes of the fitted functions for Participants 11 and 14 were notably smaller than those for the other participants. This suggests that size and pitch correspondence was not as strong for these two participants as it was for the other participants. For Experiment 2, as we sought to investigate the effect of crossmodal correspondence on variance perception, we decided to exclude Participants 11 and 14 in order to restrict the sample to those who had at least a moderate sense of crossmodal correspondence (exclusion criterion was set at less than 2 *SD* from the average value). Moreover, the participant who self-reported a problem regarding their pitch perception (Participant 20) was also excluded, although her data for Experiment 1 did not show any significant difference when compared with the data of the other participants.

Note that it is possible that the associations between size and pitch observed in Experiment 1 were cognitive and not necessarily perceptual or absolute. Crossmodal correspondence, however, may occur across various levels, such as perceptual, cognitive, and decision-making or response-selection. In the study of Gallace and Spence (2006), the link between size and pitch was considered to be relative and to involve cognitive processing, but the presentation of matching sounds was found to improve the response time in a size-classification task. Therefore, we consider that it is possible that a crossmodal link facilitates the information process, even if the link appears at the cognitive level. In Experiment 2, we investigated whether the crossmodal correspondence observed in Experiment 1 has an effect on variance perception.

## Experiment 2

In Experiment 2, we investigated whether size-pitch correspondence for each stimulus element influences the precision of associated variance discrimination. We hypothesized that the threshold of variance discrimination would decrease (increased sensitivity) when the corresponding sound is presented synchronously with each visual stimulus (crossmodal-congruent condition), because saliency and information processing would be facilitated by the crossmodally corresponding sound. However, we also hypothesized that when the combination of visual size and auditory pitch was incongruent (crossmodal-incongruent condition), this effect would not occur meaning that the sensitivity of variance discrimination would not increase. As mentioned earlier, to produce the stimuli, we applied individually tailored parameters for each participant; that is, we used different sets of audio-visual stimuli, not fixed sets, for each participant. We chose this method because the results of Experiment 1 showed that correspondence between auditory pitch and visual size depends on participants’ sensitivity to them, and the participants’ reported correspondence showed consistency. It is conceivable that using each participant’s optimal combination value is most appropriate for examining the crossmodal-congruency effect in variance perceptions.

### Methods

#### Participants

Of the 22 participants from Experiment 1, 18 participated in Experiment 2. As mentioned in the discussion of Experiment 1, three of the participants from Experiment 1 were excluded because, to fulfill the purpose of this experiment, it was necessary to possess size-pitch correspondence and normal ability regarding pitch perception. In addition, another participant (Participant 7 in Experiment 1) did not participate in Experiment 2 for private reasons.

#### Apparatuses

For Experiment 2, the same apparatuses as those used in Experiment 1 were applied.

#### Stimuli

In each trial, eight white disks were presented, one-by-one, in sequence. The disks were positioned on an outline of an invisible circle that had a radius of 1° which, to reduce the influence of any eccentricity, was located in the center of a gray background screen. The diameters of each disk were randomly chosen from the lognormal distribution lnN (lnD, *σ*^2^), having a randomly selected *baseline* diameter D between 1.0° and 1.2°. For each sequence, the baseline diameters of the two alternatives (pedestal and comparison stimuli; *pedestal* means standard stimuli) were set to differ. For the pedestal variances, two magnitudes were introduced (*σ*^2^ = 0.0484 and *σ*^2^ = 0.1156); the pedestal variance was less than that of the comparison variance. To avoid the possibility of growing tired of the participant due to the repeated presentation of the similar variance value, we introduced the two pedestal variance. The *SD*s of the comparison stimuli were determined for each trial using the QUEST algorithm (Watson & Pelli, 1983).

A sequence of eight pure tones was played through headphones and synchronized with each visual disk’s appearance. The frequency of each tone was dependent on the experimental conditions described later.

#### Experimental conditions

Four experimental conditions were set for Experiment 2: *crossmodal-congruent*, *crossmodal-incongruent*, *constant pitch*, and *no sound*. In the crossmodal-congruent condition, the frequency of each tone corresponded to each disk’s size, based on the participant’s regression equation (obtained in Experiment 1). In the crossmodal-incongruent condition, the correspondence between the visual (size) and auditory (pitch) stimuli was reversed, with eight combinations being shown; that is, combinations such as a high-pitch tone with a large circle and a low-pitch tone with a small circle, the opposite to that shown in the congruent condition, were introduced. In the constant-pitch condition, eight tones and eight circles were presented, with the respective pitches of the tones corresponding to the average sizes of the respective circles. Finally, in the no-sound condition, only visual stimuli were presented. All conditions were conducted within the same block in a random order.

#### Procedure

**Figure 2.**
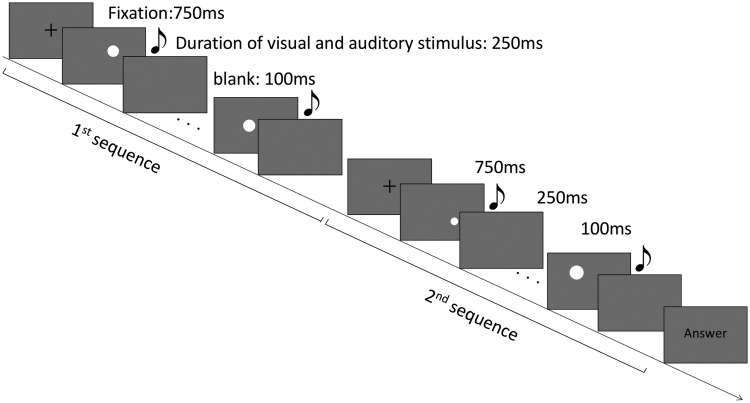
Schematic view of the stimuli used in Experiment 2. The duration of each visual and auditory stimulus was 250 ms, and the interstimulus interval was 100 ms. When auditory stimuli were presented (crossmodal-congruent, crossmodal-incongruent, and constant pitch conditions), the onset and offset times of the sounds were the same as those of the disks.

In each trial, the fixation point was shown on the screen for 750 ms, and then the first sequence (eight disks) was presented. Each disk was shown for 250 ms, with a 100-ms blank interval between each. After this, the fixation point was presented again, and then the second sequence was presented in the same way (Figure 2). When the second sequence was completed, the word *answer* was presented on the screen, and participants were required to judge which of the two successive sequences of visual stimuli had larger variance (i.e., they applied a temporal two-alternative forced choice method). Participants were instructed to focus on the size variance of the circles, and they were required to press 1 on the keyboard if the variance of the first sequence was larger, 3 if the second stimulus was larger, and 2 to proceed to the next trial. Since the trials in all four conditions were run randomly within the same block, visual stimuli were presented with accompanying auditory stimuli in some trials and without auditory stimuli in others. For those involving auditory stimuli, the onset and offset time of each sound was the same as that of the presentation of the corresponding disk. Participants were asked to ignore any sounds they heard and to judge the size variance based only on the visual stimuli. After the experiment trials, participants were asked whether they had ignored the sound and had avoided using the auditory stimuli to help them judge the size variance of the circles. There were no participants who reported having responded consciously based on the auditory stimuli.

A practice session was conducted before the experiment session. Using the QUEST algorithm (Watson & Pelli, 1983), we estimated 82% accuracy-discrimination thresholds for the eight conditions (2 pedestals × 4 crossmodal conditions). Forty-five trials were conducted for each condition, and these trials were randomly mixed in each experimental block. In total, there were 360 trials (8 Conditions × 45 Trials) and these were divided into five blocks (the QUEST program was running until the end of all five blocks; the trials were only paused between the blocks, when a blank monitor screen was shown).

### Results and Discussion

**Figure 3.**
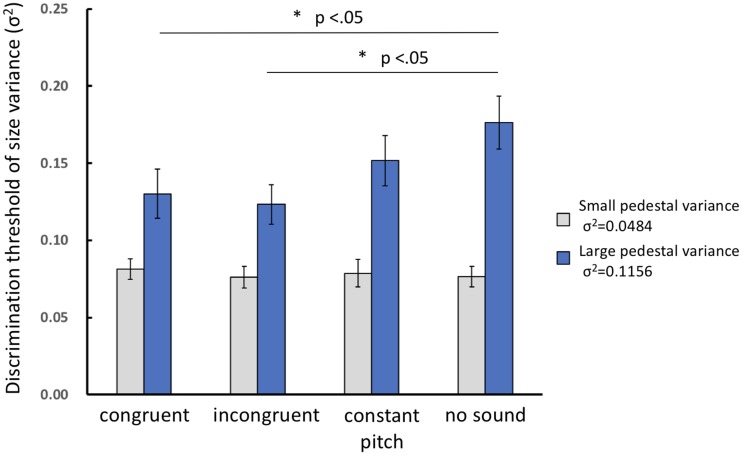
This graph shows the thresholds regarding size-variance discrimination for each condition. Light gray bars indicate small pedestal variance (*σ*^2^ = 0.0484), and dark gray bars indicate large pedestal variance (σ^2^ = 0.1156). The error bars represent the standard error (*SE*). The asterisks indicate the significant difference between the discrimination thresholds (*p* < .05 after Bonferroni correction).

Figure 3 shows the average values of the size-variance-discrimination thresholds for each condition. A two-way repeated measures analysis of variance (Pedestal Variance × the Crossmodal Condition’s Discrimination Threshold) was conducted. In the repeated measures analysis, Greenhouse-Geisser correction was used to address violations of the sphericity assumption (Geisser & Greenhouse, 1958). The main effect of pedestal variance was significant, *F*(1, 17) = 62.597, *p* < .001, but there was no significant main effect of the crossmodal condition, *F*(3, 51) = 2.764, *p* = .079; the interaction between pedestal variance and crossmodal conditions was significant, *F*(3, 51) = 4.811, *p* < .001. A post hoc test revealed that a simple main effect of crossmodal condition was significant only for the large pedestal variance (*σ*^2^ = 0.1156). For the large pedestal variance, the discrimination thresholds in the crossmodal-congruent condition (multiple comparison with Bonferroni’s method: *p* = .021 after Bonferroni correction) and in the crossmodal-incongruent condition (*p* = .033 after Bonferroni correction) were lower than those in the no-sound condition. None of the differences between the other conditions were significant.

Analysis of variance showed that there was no effect of crossmodal correspondence on small pedestal variance. This might be because the small pedestal variance was not sufficiently large for the participants to neglect the intrinsic noise. The value for the small pedestal variance that we used in this experiment was larger than that of the largest pedestal used in Solomon et al. (2011). However, in Solomon et al.’s study, all disks were presented concurrently, whereas in our study, the audio-visual stimuli were presented sequentially; thus, it is possible that noise related to memory was also added, which might have affected the variance discrimination when the external variance was not large enough. In the following discussion, we thus focus on the results for the large pedestal variance instead of the small pedestal variance.

We predicted that the discrimination threshold of the visual size variance would decrease when the audio and visual stimuli were synchronized congruently (because the correspondence of audio-visual stimuli would improve processing accuracy), but not when the audio and visual stimuli were synchronized incongruently. The results matched only a part of this prediction. The discrimination threshold in the crossmodal-congruent condition indeed decreased compared with the no-sound condition; however, contrary to the prediction, the discrimination threshold also decreased in the crossmodal-incongruent condition compared with the no-sound condition. If this increased sensitivity regarding variance discrimination had been created as a result of improved processing accuracy for the visual stimuli that were synchronized with their corresponding audio stimuli, the discrimination threshold would have been lower in the crossmodal-congruent condition than in any other condition. The result, however, differed. We will discuss possible reasons for these results in the General Discussion section.

In addition, it is notable that there was no significant difference between the constant-pitch condition and the no-sound condition. Thus, although the thresholds in both the crossmodal-congruent and crossmodal-incongruent conditions were not significantly lower than those in the constant-pitch condition, this does not mean that audio-visual co-occurrence is sufficient to explain the threshold decrements in the two crossmodal conditions. Previous studies have reported that sound has a facilitating effect on visual processing. For example, synchronous sound has been determined to facilitate the detection of flashes of light (e.g., Bolognini, Frassinetti, Serino, & Ladavas, 2005) and visual events (Noesselt, Bergmann, Hake, Heinze, & Fendrich, 2008; Vroomen & de Gelder, 2000). Furthermore, repetition blindness, which is a failure to perceive the second occurrence of a repeated item in a rapid serial visual presentation stream, has been found to decrease when sounds are synchronously presented with repeated items (Chen & Yeh, 2009). Based on the findings of the earlier studies, it could be inferred that there would be no difference between the constant-sound condition and the two crossmodal conditions (congruent and incongruent) because the presentation of constant sounds somewhat facilitates the perception of visual stimuli. Our result here, however, shows that this effect is not sufficiently large to produce a significant difference between the constant-sound and no-sound conditions. Furthermore, it means that the decrements of the thresholds in the crossmodal-congruent and crossmodal-incongruent conditions must have been caused by a mechanism related to the variance of the auditory input, not merely the existence of sound synchrony.

## General Discussion

Variance is very important information that represents the diversity of objects and groups, the reliability of information regarding these groups and, in some cases, abnormal conditions in the external world. *Consequently*, it may affect our decision-making and behaviors. In previous studies on statistical summary representation (or ensemble perception), it has been demonstrated that a human observer can perceive variance as efficiently as he or she can determine averages. Thus far, however, such findings have been limited to the domain of visual processing. As a result, we believe that the present study is the first to report on variance perception in response to multisensory information. Previous studies (e.g., Evans & Treisman, 2010; Gallace & Spence, 2006) have reported that an automatic link between different sensory modalities (e.g., high-pitch tone and small visual size, or low-pitch tone and large visual size) exists in nonsynesthetic people, and that processing of visual stimuli is facilitated when corresponding sounds are presented synchronously, even though the sound stimuli may be irrelevant to the task. Based on these results, in this study, we investigated whether such crossmodal correspondence regarding audio-visual stimuli also has a facilitating effect on variance perception. Specifically, we conducted an experiment examining whether variance-discrimination precision improves when irrelevant sounds, with pitches that correspond to the sizes of the visual stimuli, are presented synchronously.

In Experiment 1, participants were asked to resize virtual disks until they matched a corresponding sound; this was performed for five different frequencies and the results were used to determine each participant’s crossmodal-correspondence relationship. As a result, almost all participants returned a linear relationship between size and pitch; that is, the higher the pitch, the smaller the circle judged to match the sound. Further, although the values of the slopes of the regression lines differed for each participant, for most participants, the relationship between size and pitch was consistent. These results showed that the existence of consistent crossmodal correspondence between pitch and size is supported by subjective judgment, building on previous findings that observed this relationship through speeded classification tasks (Evans & Treisman, 2010; Gallace & Spence, 2006) and temporal-order judgment tasks (Parise & Spence, 2008, 2009).

In Experiment 2, using each participant’s individual crossmodal-correspondence parameters discerned in Experiment 1, the effect of crossmodal correspondence on variance perception was examined. We set four conditions: (a) a crossmodal-correspondence condition; (b) a crossmodal-incongruent condition, in which the combinations of the visual and auditory stimuli in the crossmodal-correspondence condition were reversed; (c) a constant-sound condition; and (d) a no-sound condition. The participants’ variance-discrimination precision in regard to circle sizes was compared between the conditions.

The results of Experiment 2 suggested that the presentation of a sound with a pitch that corresponds to the size of the circle shown does not affect variance-discrimination precision regarding the circles’ sizes; further, congruency between each visual and auditory stimulus was not necessary to improve variance discrimination. Instead, in the large-pedestal-variance condition, variance-discrimination precision improved in both the crossmodal-congruent and crossmodal-incongruent conditions compared with the no-sound condition. Why did this happen? One possibility is that, for both the congruent and incongruent conditions, the simultaneous presentation of visual and auditory stimuli with the same variance increased the size of the sample the participants could accommodate. Previous studies have shown that the number of samples that humans can accommodate in ensemble perception is limited (Ariely, 2001; Chong & Treisman, 2005; Haberman et al., 2009; Myczek & Simons, 2008; Piazza et al., 2013), so it is fully conceivable that all eight elements were not necessarily sampled in Experiment 2. In this case, if some sets from the audio-visual elements were sampled as pairs based on their occupying the same points in a stimuli sequence, and if the element values of multiple modalities are represented on a common scale with regard to variance perception, the size of the sample would have increased (up to double) in comparison to a single-modality case. Thus, in the congruent and incongruent conditions, there is the possibility that the above conditions were satisfied, consequently improving the variance-discrimination precision. (Because the scale of visual and auditory stimuli is different, there is room for discussion about whether the magnitudes of variance might be equal. However, at least the magnitude of the variances of two successive sequences of stimuli has the same degree of correlation between visual and auditory modalities.)

It is conceivable that the improvement in processing accuracy (including saliency) for each stimulus facilitated the overall variance perception. In this study, we hypothesized that the processing accuracy of individual stimuli would improve when audio-visual stimuli with a crossmodal link were presented concurrently, but this effect was not observed in our results. In contrast, Gallace and Spence (2006) found, in a speeded discrimination task, that presenting an irrelevant sound that corresponded to the size of visual stimuli improved reaction time. The reason for the difference between the results of our study and those of Gallace and Spence (2006) could be that while crossmodal correspondence is effective in regard to facilitating perceptual processing and accelerating decisions, it may not change the internal quality of the perceptual stimuli. In fact, Gallace and Spence (2006) also reported, after analysis of the point of subjective equality for the size-discrimination task, that the presentation of the sound had no effect on the perceived size of the disks, despite its significant effect on response latencies.

However, we consider that our hypothesis regarding improvement in processing accuracy was not completely rejected. It is possible that there were other causes of the apparent identical threshold decrement between the crossmodal-congruent and crossmodal-incongruent conditions. Processing accuracy might have been improved in the crossmodal-congruent condition, but the sample size increment in this condition might not have been as large as that in the crossmodal-incongruent condition; therefore, the total variance-discrimination promotion effect in the two conditions might have been equivalent. A previous study using a crossmodal temporal order judgment task demonstrated that the stronger the synesthetic coupling between the pitch and the size, the more difficult it is to perceive the spatiotemporal deviation (asynchrony) between them (Parise & Spence, 2008, 2009). This suggests that multisensory integration is facilitated by crossmodal correspondence between modalities. Thus, it is conceivable that in the crossmodal-congruent condition, the fusion of audio-visual stimuli was promoted, and the separability of each modality decreased. For this reason, the sample size in the variance discrimination possibly did not increase. The above possibility is mere speculation, of course, and further investigation is necessary.

The effect of multisensory input on variance perception may differ depending on the situation. In this experiment, the auditory stimuli were presented as irrelevant to the task. If auditory stimuli are recognized as information generated from the same source (e.g., a human face and a human voice), the variance-perception precision may improve. Further, when the noise of the individual stimulus is larger (e.g., short presentation time), or when there are stronger links between the visual and auditory stimuli, the multisensory information might be more effective. The promotion effect of corresponding audio-visual stimuli has been shown in a wide range of tasks, such as visual learning of motion (Kim, Seitz, & Shames, 2008) and emotional response (e.g., de Gelder & Vroomen, 2000; Kreifelts, Ethofer, Grodd, Erb, & Wildgruber, 2007). It follows that it is necessary to investigate whether the crossmodal link between stimuli can help individuals use various stimuli to understand the meaning of the summary statistics they acquire and apply this to the entire set of stimuli. This approach could reveal the characteristics of perceptions of multisensory variance.

To date, no research has been conducted on variance perception regarding information derived from multiple modalities. However, variance-perception processing does not occur independently within a single modality; there is a possibility that some common processing exists between the visual and auditory modalities. In the experiments described in this study, although participants were instructed to ignore the auditory stimuli, the variation of the pitches of the tones might have been automatically sampled and pooled with the sizes of the visual stimuli, which may have influenced the perceived size variance. Variance is a unique statistical index that differs from averaging, in that the magnitude of variance can be compared, to some extent, among different stimulus attributes and sensory modalities. Therefore, it is conceivable that there are common mechanisms underlying the perception of the variance of different attributes and sensory modalities. Further investigation of this issue through the use of different experimental methods, such as learning transition and after-effects among different sensory stimuli, are needed.

## Declaration of Conflicting Interests

The author(s) declared no potential conflicts of interest with respect to the research, authorship, and/or publication of this article.

## Funding

The author(s) disclosed receipt of the following financial support for the research, authorship, and/or publication of this article: This work was supported by JSPS KAKENHI Grant Number 15H03462.

## References

[bibr1-2041669518815709] Albrecht A. R. Scholl B. J. (2010) Perceptually averaging in a continuous visual world: Extracting statistical summary representations over time. Psychological Science 21: 560–567. doi:10.1167/9.8.957. 20424102 10.1177/0956797610363543

[bibr2-2041669518815709] Albrecht A. R. Scholl B. J. Chun M. M. (2012) Perceptual averaging by eye and ear: Computing summary statistics from multimodal stimuli. Attention, Perception & Psychophysics 74: 810–815. doi:10.3758/s13414-012-0293-0. 10.3758/s13414-012-0293-0 22565575

[bibr3-2041669518815709] Alvarez G. A. (2011) Representing multiple objects as an ensemble enhances visual cognition. Trends in Cognitive Sciences 15: 122–131. doi:10.1016/j.tics.2011.01.003. 21292539 10.1016/j.tics.2011.01.003

[bibr4-2041669518815709] Ariely D. (2001) Seeing sets: Representation by statistical properties. Psychological Science 12: 157–162. doi:10.1111/1467-9280.00327. 11340926 10.1111/1467-9280.00327

[bibr5-2041669518815709] Ashitani, Y., Yakushijin, R., & Iahiguhi, A. (2012, May). *Variance discrimination of empty time intervals marked by auditory tone*. Poster session presented at the Association for Psychological Science 24th Annual Convention, Chicago, USA.

[bibr6-2041669518815709] Attarha M. Moore C. M. Vecera S. P. (2014) Summary statistics of size: Fixed processing capacity for multiple ensembles but unlimited processing capacity for single ensembles. Journal of Experimental Psychology: Human Perception & Performance 40: 1440–1449. doi:10.1037/a0036206. 24730736 10.1037/a0036206 PMC7017936

[bibr7-2041669518815709] Bauer B. (2009) Does Stevens’s power law for brightness extend to perceptual brightness averaging? The Psychological Record 59: 171–186. doi:10.1007/BF03395657.

[bibr8-2041669518815709] Bolognini N. Frassinetti F. Serino A. Làdavas E. (2005) “Acoustical vision” of below threshold stimuli: Interaction among spatially converging audiovisual inputs. Experimental Brain Research 160: 273–282. doi:10.1007/s00221-004-2005-z. 15551091 10.1007/s00221-004-2005-z

[bibr9-2041669518815709] Brainard D. H. (1997) The psychophysics toolbox. Spatial Vision 10: 433–436. 9176952

[bibr10-2041669518815709] Chen Y. C. Yeh S. U. (2009) Catch the moment: Multisensory enhancement of rapid visual events by sound. Experimental Brain Research 198: 209–219. doi:10.1163/156856897X00357. 19444433 10.1007/s00221-009-1831-4

[bibr11-2041669518815709] Chong S. C. Treisman A. (2003) Representation of statistical properties. Vision Research 43: 393–404. doi:10.1016/S0042-6989(02)00596-5. 12535996 10.1016/s0042-6989(02)00596-5

[bibr12-2041669518815709] Chong S. C. Treisman A. (2005) Attentional spread in the statistical processing of visual displays. Perception & Psychophysics 67: 1–13. doi:10.3758/BF03195009. 15912869 10.3758/bf03195009

[bibr13-2041669518815709] de Gelder B. Vroomen J. (2000) The perception of emotions by ear and by eye. Cognition and Emotion 14: 289–311. doi:10.1080/026999300378824.

[bibr14-2041669518815709] Emmanouil T. A. Treisman A. (2008) Dividing attention across feature dimensions in statistical processing of perceptual groups. Perception & Psychophysics 70: 946–954. doi:10.3758/PP.70.6.946. 18717382 10.3758/pp.70.6.946

[bibr15-2041669518815709] Ernst M. O. Buülthoff H. H. (2004) Merging the senses into a robust percept. Trends in Cognitive Sciences 8: 162–169. doi:10.1016/j.tics.2004.02.002. 15050512 10.1016/j.tics.2004.02.002

[bibr16-2041669518815709] Evans K. K. Treisman A. (2010) Natural cross-modal mappings between visual and auditory features. Journal of Vision 10: 6.1–12 doi:10.1167/10.1.6. 10.1167/10.1.6 PMC2920420 20143899

[bibr17-2041669518815709] Gallace A. Spence C. (2006) Multisensory synesthetic interactions in the speeded classification of visual size. Perception & Psychophysics 68: 1191–1203. doi:10.3758/BF03193720. 17355042 10.3758/bf03193720

[bibr18-2041669518815709] Geisser S. Greenhouse S. W. (1958) An extension of Box’s results on the use of the F distribution in multivariate analysis. The Annals of Mathematical Statistics 29: 885–891. doi:10.1214/aoms/1177706545.

[bibr19-2041669518815709] Haberman J. Harp T. Whitney D. (2009) Averaging facial expression over time. Journal of Vision 9: 1.1–13 doi:10.1167/9.11.1. 10.1167/9.11.1 PMC2857387 20053064

[bibr20-2041669518815709] Haberman J. Lee P. Whitney D. (2015) Mixed emotions: Sensitivity to facial variance in a crowd of faces. Journal of Vision 15: 16, doi:10.1167/15.4.16. 10.1167/15.4.16 26676106

[bibr21-2041669518815709] Haberman J. Whitney D. (2007) Rapid extraction of mean emotion and gender from sets of faces. Current Biology 17: R751–R753. doi:10.1016/j.cub.2007.06.039. 17803921 10.1016/j.cub.2007.06.039 PMC3849410

[bibr22-2041669518815709] Haberman J. Whitney D. (2009) Seeing the mean: Ensemble coding for sets of faces. Journal of Experimental Psychology: Human Perception and Performance 35: 718–734. doi:10.1037/a0013899. 19485687 10.1037/a0013899 PMC2696629

[bibr23-2041669518815709] Kim R. S. Seitz A. R. Shams L. (2008) Benefits of stimulus congruency for multisensory facilitation of visual learning. PLoS One 1: e1532, doi:10.1371/journal.pone.0001532. 10.1371/journal.pone.0001532 PMC2211398 18231612

[bibr24-2041669518815709] Kreifelts B. Ethofer T. Grodd W. Erb M. Wildgruber D. (2007) Audiovisual integration of emotional signals in voice and face: An event-related fMRI study. Neuroimage 37: 1445–1456. 17659885 10.1016/j.neuroimage.2007.06.020

[bibr25-2041669518815709] Maeda F. Kanai R. Shimojo S. (2004) Changing pitch induced visual motion illusion. Current Biology 14: R990–R991. doi:10.1016/j.cub.2004.11.018. 15589145 10.1016/j.cub.2004.11.018

[bibr26-2041669518815709] Marks, L. E. (1987). On cross-modal similarity: Auditory–visual interactions in speeded discrimination. *Journal of Experimental Psychology: Human Perception and Performance*, *13*, 384–394. DOI: 10.1037/0096-1523.13.3.384. 10.1037//0096-1523.13.3.384 2958587

[bibr27-2041669518815709] Martino, G., & Marks, L. E. (1999). Perceptual and linguistic interactions in speeded classification: Tests of the semantic coding hypothesis. *Perception*, *28*, 903–923. doi:10.1068/p2866. 10.1068/p2866 10664781

[bibr28-2041669518815709] Melara R. D. O’Brien T. P. (1987) Interaction between synesthetically corresponding dimensions. Journal of Experimental Psychology: General 116: 323–336. doi:10.1037/0096-3445.116.4.323.

[bibr29-2041669518815709] Morgan M. Chubb C. Solomon J. A. (2008) A “dipper” function for texture discrimination based on orientation variance. Journal of Vision 8: 1–9. doi:10.1167/8.11.9. 10.1167/8.11.9 PMC4135071 18831603

[bibr30-2041669518815709] Myczek K. Simons D. J. (2008) Better than average: Alternatives to statistical summary representations for rapid judgments of average size. Perception & Psychophysics 70: 772–788. doi:10.3758/PP.70.5.772. 18613626 10.3758/pp.70.5.772

[bibr31-2041669518815709] Noesselt T. Bergmann D. Hake M. Heinze H. J. Fendrich R. (2008) Sound increases the saliency of visual events. Brain Research 1220: 157–263. doi:10.1016/j.brainres.2007.12.060. 18234167 10.1016/j.brainres.2007.12.060

[bibr32-2041669518815709] Parise C. Spence C. (2008) Synesthetic congruency modulates the temporal ventriloquism effect. Neuroscience Letters 442: 257–261. doi:10.1016/j.neulet.2008.07.010. 18638522 10.1016/j.neulet.2008.07.010

[bibr33-2041669518815709] Parise C. Spence C. (2009) “When birds of a feather flock together”: Synesthetic correspondences modulate audiovisual integration in non-synesthetes. PLoS One 4: e5664, doi:10.1371/journal.pone.0005664. 19471644 10.1371/journal.pone.0005664 PMC2680950

[bibr34-2041669518815709] Parkes L. Lund J. Angelucci A. (2001) Compulsory averaging of crowded orientation signals in human vision. Nature Neuroscience 4: 739–744. doi:10.1038/89532. 11426231 10.1038/89532

[bibr35-2041669518815709] Pelli D. G. (1997) The VideoToolbox software for visual psychophysics: Transforming numbers into movies. Spatial Vision 10: 437–442. doi:10.1163/156856897X00366. 9176953

[bibr36-2041669518815709] Piazza E. A. Sweeny T. D. Wessel D. Silver M. A. Whitney D. (2013) Humans use summary statistics to perceive auditory sequences. Psychological Science 24: 1389–1397. doi:10.1371/10.1177/0956797612473759. 23761928 10.1177/0956797612473759 PMC4381997

[bibr37-2041669518815709] Ramachandran V. S. Hubbard E. M. (2001) Synaesthesia—A window into perception, thought and language. Journal of Consciousness Studies 8: 3–34.

[bibr38-2041669518815709] Ramachandran V. S. Hubbard E. M. (2003) Hearing colors, tasting shapes. Scientific American 288: 52–59. 10.1038/scientificamerican0503-52 12701330

[bibr39-2041669518815709] Robitaille N. Harris I. M. (2011) When more is less: Extraction of summary statistics benefits from larger sets. Journal of Vision 11: 18, doi:10.1167/11.12.18. 10.1167/11.12.18 22031908

[bibr40-2041669518815709] Solomon J. A. (2010) Visual discrimination of orientation statistics in crowded and uncrowded arrays. Journal of Vision 10: 19, doi:10.1167/10.14.19. 10.1167/10.14.19 21163954

[bibr41-2041669518815709] Solomon J. A. Morgan M. Chubb C. (2011) Efficiencies for the statistics of size discrimination. Journal of Vision 11: 13, doi:10.1167/11.12.13. 10.1167/11.12.13 PMC4135075 22011381

[bibr42-2041669518815709] Spence C. (2011) Crossmodal correspondences: A tutorial review. Attention, Perception & Psychophysics 73: 971–995. doi:10.3758/s13414-010-0073-7. 10.3758/s13414-010-0073-7 21264748

[bibr43-2041669518815709] Ueda S. Yakushijin R. Ishiguchi A. (2015) Ability to perceive operation-response noise and the decision to stop system operation. The Japanese Journal of Psychology 86: 121–131. 26182488 10.4992/jjpsy.86.14004

[bibr44-2041669518815709] Vroomen J. de Gelder B. (2000) Sound enhances visual perception: Cross-modal effects of auditory organization on vision. Journal of Experimental Psychology: Human Perception & Performance 26: 1583–1590. doi:10.1037/0096-1523.26.5.1583. 11039486 10.1037//0096-1523.26.5.1583

[bibr45-2041669518815709] Watamaniuk S. N. Duchon A. (1992) The human visual system averages speed information. Vision Research 32: 931–941. doi:10.1016/0042-6989(92)90036-I. 1604862 10.1016/0042-6989(92)90036-i

[bibr46-2041669518815709] Watamaniuk S. N. Sekuler R. Williams D. W. (1989) Direction perception in complex dynamic displays: The integration of direction information. Vision Research 29: 47–59. doi:10.1016/0042-6989(89)90173-9. 2773336 10.1016/0042-6989(89)90173-9

[bibr47-2041669518815709] Watson A. B. Pelli D. G. (1983) QUEST: A Bayesian adaptive psychometric method. Perception & Psychophysics 33: 113–120. doi:10.3758/BF03202828. 6844102 10.3758/bf03202828

[bibr48-2041669518815709] Weiss D. J. Anderson N. H. (1969) Subjective averaging of length with serial presentation. Journal of Experimental Psychology 82: 52–63. doi:10.1037/h0028028.

